# Differential morphology and transcriptome profile between the *incompletely fused carpels* ovary and its wild-type in maize

**DOI:** 10.1038/srep32652

**Published:** 2016-09-02

**Authors:** Hongping Li, Yufeng Wu, Yali Zhao, Xiuli Hu, Jianfeng Chang, Qun Wang, Pengfei Dong, Moubiao Zhang, Chaohai Li

**Affiliations:** 1 Agronomy College, Collaborative Innovation Center of Henan Grain Crops, Henan Agricultural University, Zhengzhou 450002, P. R. China; 2 Bioinformatics Center, National Key Laboratory of Crop Genetics and Germplasm Enhancement, Nanjing Agricultural University, Nanjing 210095, P. R. China

## Abstract

We have isolated a new mutation in maize, *incompletely fused carpels* (*ifc*), which results in an open stylar canal on the ovary and an incomplete pericarp at the top of the kernel. The maize ovary derives from the fusion of three carpels; however, the molecular networks regulating maize carpel fusion remain largely unclear. In this study, RNA sequencing (RNA-seq) was performed on wild-type (WT) and *ifc* ovaries that were collected after carpel fusion defects could be morphologically distinguished. In total, 877 differentially expressed genes were identified. Functional analysis revealed overexpression of genes related to “DNA binding”, “transcription regulation”, “hormones”, and “stress responses”. Among the 88 differentially expressed transcription factor (TF) genes, five showed a high degree of conservation (77.7–88.0% amino acid identity) of their conserved domains with genes associated with carpel fusion deficiency in *Arabidopsis thaliana*, suggesting that these five genes might control carpel fusion in maize. In addition, 30 genes encoding components of hormone synthesis and signaling pathways were differentially expressed between *ifc* and WT ovaries, indicating complex hormonal regulation during carpel fusion. These results help elucidate the underlying mechanisms that regulate carpel fusion, supporting the functional analysis of genes involved in producing this phenotype.

The plant ovary is derived from carpels, and its formation requires the coordinated growth and development of carpel marginal tissue, which is of critical importance for fruit shape, seed dispersal, and grain yield. The molecular regulation of carpel development relies on a complex regulatory pathway involving microRNA genes, transcription factors (TFs), and hormonal components^1^.

The carpel, which harbors and promotes the fertilization of ovules, is a unit structure of the gynoecium. The gynoecium is a structure formed from the fusion of one or more carpels that constitutes the inner whorl of the floral organ. In cereals, the gynoecium, which is composed of the ovule and ovary wall, develops into the kernel. In maize, the female inflorescence or the ear initiates florets composed of four types of floral organs: the palea, lodicule, stamen, and pistil. Each pistil develops into a single fruit, the kernel. At maturity, the maize ear is an aggregation of fruits, and each of the fruits is a child or a seed. The maternally derived ovary wall of the kernel undergoes morphological changes and develops into a mature pericarp, which completely encloses the seed^2^. In maize, the gynoecium is formed from three carpels by congenitally fusing their edges, with two indeterminate abaxial carpels fusing to form the silk, and the third elongating only enough to cover the ovule^2^,^3^. The stylar canal is formed at the point where the adaxial carpel and the two abaxial carpels meet^4^. The stylar canal represents the only route from outside of the ovary to the ovary cell walls^5^. Defects in the stylar canal result in aberrant ovary development and susceptibility of the kernel to pathogenic infections, which have serious consequences for seed vigor and grain yield^6^. Kernels with a closed stylar canal are used for selecting maize lines that exhibit increased resistance to diseases^5^.

Previous studies have revealed a complex functional hierarchy of genes involved in carpel development in the model plant *Arabidopsis* (*Arabidopsis thaliana*)^7^. Carpel identity is specified by at least two separate pathways: one is mediated by the class C gene *AGAMOUS* (*AG*)^8^, and class E or *SEPALLATA* genes, as described in the expanded ABC model^9^; the second is an AG-independent pathway involving *SPT* and *CRC* genes^10^, both of which are negatively regulated by class A genes^11^, such as *APETALA2* (*AP2*) and *LEUNIG* (*LUG*)^12^,^13^,^14^. In addition, the *Arabidopsis thaliana CUP-SHAPED COTYLEDON* (*AtCUC2*) gene, together with *AtCUC1*, is required for the specification of the carpel boundary primordium^15^. Overexpression of *AtCUC2* can prevent congenital carpel fusion^16^. Furthermore, microRNA164, which mediates the repression of *AtCUC1* and *AtCUC2*, is necessary for organ boundary formation^17^. The development of the valve or carpel margin lays the very early foundation for gynoecium development^1^. Previous studies focused on the determination of carpel organ identity and carpel margin differentiation, but the development and regulation of carpel fusion remains less clear.

In maize (*Zea mays* L.), much less is known than in *Arabidopsis* about the gene regulatory networks that regulate the differentiation and determine the congenital fusion of the carpels. This is because there are very few known mutations with a phenotype restricted to the carpel, although some aspects of carpel tissue development are conserved in *Arabidopsis*. For example, mutations in maize homologs of the *Arabidopsis* class C gene *AG* result in extra carpels that fail to fuse into functional silk in the *zag1* mutant, suggesting that at least some class C genes that function in carpel identity are conserved^18^,^19^. In maize, the homeobox gene *knotted1* (*kn1*) is also required for female floret development. Mutations in *kn1* result in extra carpels with proliferating ovule tissue^4^. These genes provide a framework for the molecular regulation of pistil development in maize and indicate the existence of distinct genes and regulatory programs restricted to carpel development. Thus, genes need to be identified and characterized more precisely to understand exactly how carpel tissue morphologies are specified in maize.

The concerted action of multiple hormones is required for the control of carpel development. In particular, genes involved in the auxin pathway play a key role in the regulation of carpel development and gynoecium patterning^20^,^21^, and there is increasing evidence of involvement of auxin biosynthesis and signaling in floral tissue development in *Arabidopsis*^22^. However, a specific description of auxin-related gene expression associated with carpel fusion is currently lacking. Moreover, other hormones, including gibberellin (GA)^23^, cytokinin (CK)^24^, and brassinosteroids (BRs)^25^, have been implicated in *Arabidopsis* carpel development, but detailed knowledge of their activity and related patterns of gene expression associated with maize carpel fusion are limited.

Despite recent advances in the regulation of flower development and ovary formation^1^, the mechanisms by which transcriptional regulators and hormonal activity interact to coordinate the growth and differentiation of carpel fusion remain poorly understood. Although previous studies analyzing carpel mutations have used forward genetic methods to screen genes, they have typically lacked mapping and quantification of the overall transcriptome to uncover biological pathways and regulatory networks^26^,^27^,^28^.

In this study, we conducted morphological and histological surveys to capture the progressive stages of carpel development. Moreover, we performed RNA-seq analysis to investigate transcriptional networks that are likely to regulate *ifc* ovary formation. The aims of this study were to understand carpel fusion in the maize ovary and to identify significant differences in the transcriptome between the *ifc* ovary and its wild-type (WT). Maize exhibits a similar floret morphology to a typical dicot flower, along with the conserved genetic mechanism underlying flower development, and is therefore suitable for use in studies investigating reproductive organ developmental^2^. Here, we report a preliminary atlas of the transcriptome characteristics of *ifc* and wild-type ovaries to begin to decipher carpel development and, ultimately, to provide fundamental insight into the underlying mechanisms that determine ovary formation, grain yield, and seed quality.

## Results

### Morphological and Histological Analysis of *ifc* Ovary Development

**Figure 1:**
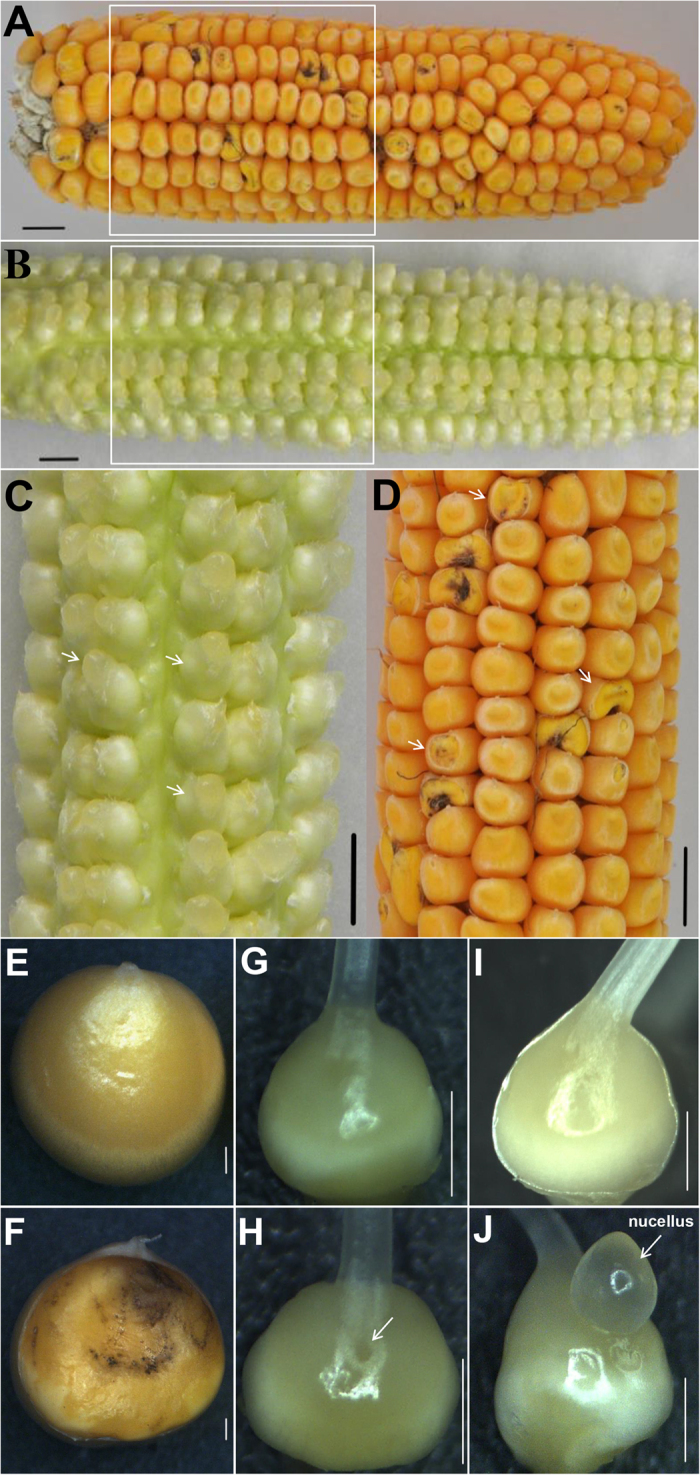
Phenotypes of the WT and *ifc* kernels showing an incomplete ovary wall. (**A,D**) Mature ear with WT and *ifc* kernels. (**B,C**) Ear at 2 days after silking; *ifc* ovaries were interspersed among WT ovaries, and are indicated by arrows in (**C,D). (E**) Mature WT kernels with an intact pericarp. (**F**) Mature *ifc* kernels without a pericarp wrapping at the top. (**G** to **J**) Structure of the WT and *ifc* pistils. (**H)** A *ifc* pistil with a small carpel rupture (indicated with an arrow) that is just becoming observable. (**J**) A *ifc* pistil at silking with a protruding nucellus (indicated by the arrow). (**G,I**) WT pistils in the same period as those in H and J, respectively. Bars = 1 cm (**A** to **D**) and 1 mm (**E** to **J**). WT, wild-type; *ifc*, *incompletely fused carpels*.

**Figure 2:**
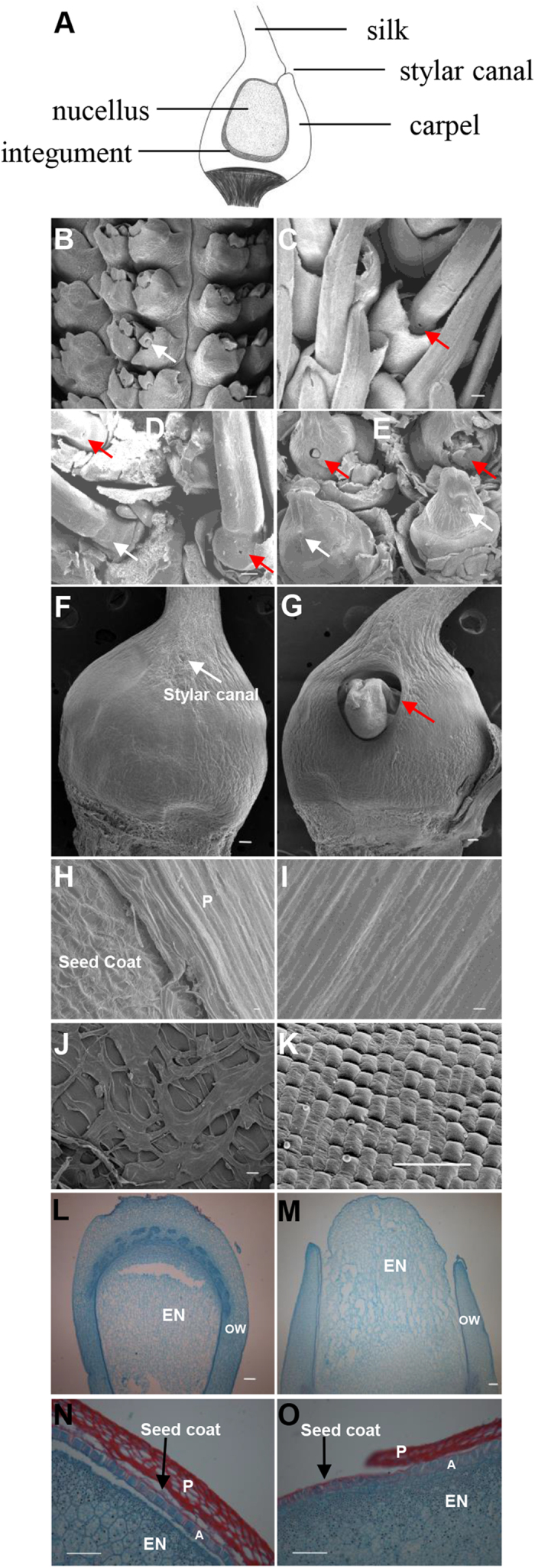
SEM and histological micrographs depicting the development of WT and *ifc* ovaries and kernels. (**A**) Diagram of a cross-section through a developing maize ovary. (**B**,**C**) Two stages of ear development. (**B**), A 1 cm-long portion of an ear. (**C**) A 4 cm-long portion of an ear. (**D**,**E**) Anatomical structure of the pistils with the glumes removed in 4 cm- and 6 cm-long ears, respectively. (**F,G**) Close-up views of WT and *ifc* ovaries in 8 cm-long ears, respectively. (**H** to **K**), Histological features of the maize kernel surface in WT (**I**,**K**) and *ifc* (**H**,**J**). (**H**) Surface of an *ifc* kernel showing the transition from the pericarp to the seed coat. (**I**) Surface of a WT kernel. (**J**) Close-up view of the seed coat in (**H**) without pericarp packaging. (**K**) The surface of the aleurone layer with the cells arranged neatly in the WT kernel in DAP 15. The outer pericarp was removed. (**L** to **O**) Longitudinal section through WT (**L**,**N**) and *ifc* (**M**,**O**) ovaries at DAP 3 (**L**,**M**) and DAP 30 (**N**,**O**), showing an incomplete pericarp (P) and an exposed seed coat outside the aleurone layer (A) in *ifc* mutants, versus an intact pericarp and seed coat wrapping in WT. The normal stylar canal is indicated by white arrows in (**D–F**) and the unfused stylar canal is indicated by red arrows in (**D**,**E**,**G**). DAP, days after pollination; WT, wild-type; *ifc*, *incompletely fused carpels*; OW, ovary wall; EN, endosperm; P, pericarp; A, aleurone layer; Si, silk. Bars = 1 mm (**B** to **G**,**H**,**L**,**M**) and 100 μm (**I** to **K**,**N**,**O**).

To understand the molecular regulation of carpel fusion during maize ovary development, we characterized the *ifc* phenotype of YU-A474 (an inbred line) in detail. This phenotype was discovered based on a mature kernel (Fig. 1A,D–F) with a gap on the top, which was close to the silk and back to the embryo (Fig. 1F). Careful examination of many ears showed that the *ifc* phenotype of the pistil arose before pollination (Fig. 1B,C,G–J), and the gap at the top of the *ifc* ovary was observed ahead of the extruding nucellus (Fig. 1H,J). It was also found that there was no significant difference in silk length between the *ifc* ovaries and their wild-type (Table S1). The ovaries of *ifc* pistils were normally enough to satisfy pollination and fertilization (Fig. 1F; Figure S1). There were no detectable abnormalities in other organs. The pistil, which is the inner whorl of the floret, begins as a ridge on the abaxial surface of the apical meristem. Continued overgrowth of ridge tissue initiates silk development and leads to the formation of the stylar canal (Fig. 2A).

Initially, some ears exhibited a few kernels with a gap at the top at maturity, and the phenotype was very weak. After additional generations of self-pollination, a strong phenotype was observed. However, a maximum of 80% of the kernels from the self-crossed *ifc* progenies displayed an incomplete pericarp, and the remaining kernels showed an intact pericarp, indicating partial penetrance of the *ifc* phenotype. To determine whether *ifc* is a dominant or recessive mutation, F_1_ ears from two reciprocal crosses of plants were investigated. We did not observe any F_1_ ears with the *ifc* phenotype (Table S2). In addition, 831 ears of BC_1_ plants and 790 ears of F_2_ plants showed 1.10:1 (χ^2^ = 2.02; χ^2^_0.05, 1_ = 3.84; χ^2 ^< χ^2^_0.05, 1_) and 2.67:1 (χ^2^ = 2.07; χ^2^_0.05, 1_ = 3.84; χ^2 ^< χ^2^_0.05, 1_) segregation, respectively (Table S2). This genetic analysis suggested that *ifc* is a recessive allele.

We examined the ears, which represent a range of developmental stages from 5 to 70 mm in length, via SEM. Early ear development preceded normally (Fig. 2B), and no defects were observed in the branch meristem, spikelet pair meristem, or spikelet meristem (data not shown). An aberrant phenotype first became apparent in the ears when they were approximately 60 mm long (Fig. 2C,D) at which time they were sufficiently well developed to exhibit some florets with incompletely fused carpels, distinguished by an open stylar canal, rather than the completely fused carpels observed in the WT (Fig. 2D,E). This phenotype was particularly prominent in the more advanced ears (>60 mm) (Fig. 2E–G), especially after pollination. The *ifc* ovary exhibited an incomplete kernel surface with a distinct transition from the pericarp to the seed coat (Fig. 2H), which was completely wrapped inside in the WT kernel (Fig. 2I). In the *ifc* maize kernel, the seed coat was exposed on the outside (Fig. 2J), without a covering. Additionally, the aleurone was neatly arranged in a one-celled layer in the *ifc* kernel (Fig. 2K).

Then, we performed histological examination of the kernels after pollination to examine the changes in the kernel surface structure. It was found that the seed coat gradually became a thin membrane between the aleurone and the pericarp and was reduced to a thin remnant in both the WT and *ifc* kernels (Fig. 2L–O). While the arrangement of the aleurone layer was not so neat in the *ifc* kernels as in the WT kernels, it was constrained only by the seed coat (Fig. 2N,O). Serial observations from pistil formation to the mature kernel further confirmed that there was no ovary wall/pericarp on the surface of the gap.

### Mapping and Analysis of Transcriptome Sequence Data

To compare the transcriptomes of *ifc* ovaries and WT ovaries, we conducted high-throughput RNA-seq using the Illumina HiSeq-2500 platform. Ovaries were collected when the *ifc* phenotype first became apparent (Fig. 1H). We selected this stage because early floral organogenesis could not be identified to deviate from normal development, and at later stages, the aberrant phenotype became particularly prominent and was farther away from the point of complete carpel fusion in the WT ovary (Figure S1). Three biological replicates for both phenotypes were collected for the production of RNA-seq libraries.

**Figure 3:**
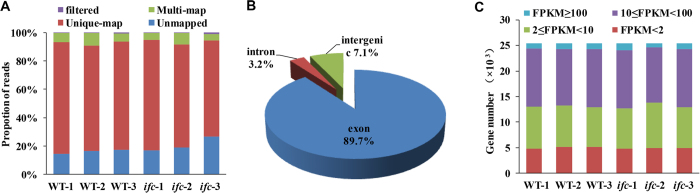
Analysis of sequenced RNAs from WT and *ifc* ovaries when the incompletely fused carpels were just becoming observable. (**A**) Statistics for the raw reads after being filtered and aligned to the B73 v3 reference genome. (**B**) Distribution of the reads among maize reference genomic features. (**C**) Number of genes showing different expression levels (based on FPKM) in each sample. WT, wild-type; *ifc*, *incompletely fused carpels.* Biological replicates are indicated as −1, −2, and −3.

Sequencing resulted in an average of 52 million paired-end reads (125-bp read length) for each sample (Figure S2A; Table S3). We checked the quality of the generated reads and mapped them to the maize reference genome (ZmB73_RefGen_v3)^29^ (Table S3). Among the resulting mapped reads, an average of 81.4% of the reads mapped to a unique position in the genome (Fig. 3A), and 89.7% of the reads mapped to exonic sequences (Fig. 3B). Moreover, among the reads that mapped to splice events and splice junctions, 94.8% and 76.3%, respectively, were annotated (Figure S2B,C). The genomic distribution of the RNA-seq reads demonstrated that the majority of the detected genes were predicted in the maize annotation.

The exonic reads were then subjected to Cufflinks assembly^30^ and reported as fragments per kilobase of transcript per million mapped reads (FPKM)^31^. A gene was considered to be expressed in a given sample if the lower boundary of its FPKM 95% confidence interval (FPKM_conf_lo) was greater than zero^32^. Based on this criterion, we identified a total of 35,905 (90.6% of total) genes that were expressed in at least one of the six samples (Table S4). Pairwise Spearman correlation coefficient (SCC) analysis showed that the triplicate FPKM values from both the WT and *ifc* ovaries were highly correlated (r = 0.96 to 0.98; Figure S3), indicating high reproducibility of the RNA-seq experiments. The proportions of genes exhibiting high expression (FPKM ≥ 10), intermediate expression (2 ≤ FPKM < 10), and low expression (FPKM < 2) were relatively similar in the two phenotypes (Fig. 3C; Table S5). There was little variation in the total number and distribution of the abundance of genes represented in the two phenotypes, similar to other transcriptomic studies on ear development mutant^33^. Taken together, our analysis of transcriptome sequence data indicates that we obtained sufficient coverage of the mRNA population to distinguish the functional complexity of gene networks associated with carpel fusion.

### The *ifc* and WT Ovaries Exhibit Distinct mRNA Populations

We employed Cuffdiff^30^ to identify differentially expressed genes, using the following criteria: fold change >1.5 between WT and *ifc*, *p* value < 0.05, and FPKM value > 2 in at least one of the samples (Table S6). This analysis identified a total of 877 differentially expressed genes between the two phenotypes (Table S7), representing 3.5% of the ovary expressed genes (25,369) identified in the two phenotypes. Because the WT and *ifc* mutant ovaries came from the same ear, this indicated that most of the detected genes displayed similar expression levels between WT and *ifc*. Compared with WT, 592 genes were up-regulated in *ifc* mutant ovaries, and 285 genes were down-regulated (Table S7). We detected a small fraction of transcripts derived from transposable elements (TEs) annotated in release 5b.60, with an average of 2,996 expressed TEs in both phenotypes (Table S8).

**Figure 4:**
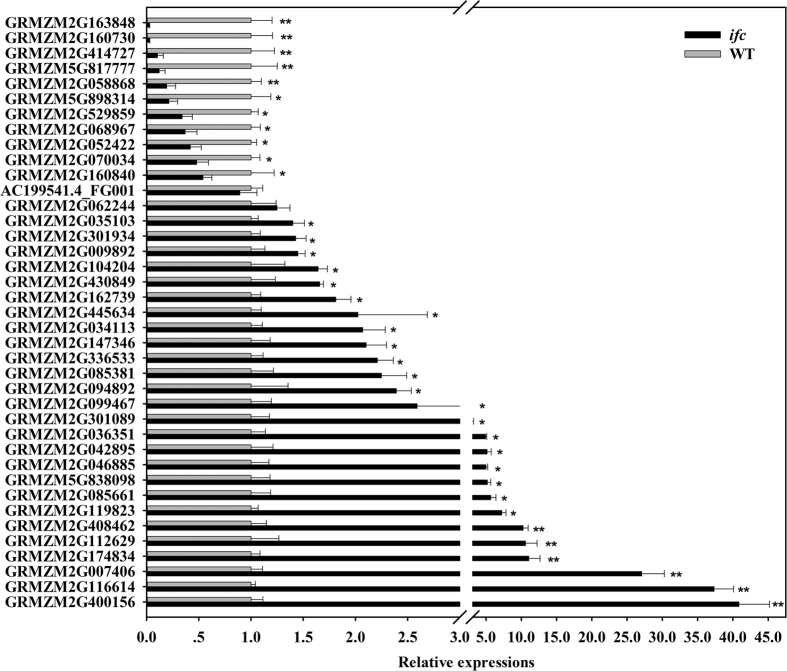
Quantitative RT-PCR results for all 39 validated genes. For information on the primers used to evaluate these genes through qRT-PCR, see Figure S12. “//” indicates 3–3.1. Error bars represent the standard error (SD). Values are means ± SD. WT, wild-type; *ifc*, *incompletely fused carpels*. *P value < 0.05; **P value < 0.01.

**Figure 5:**
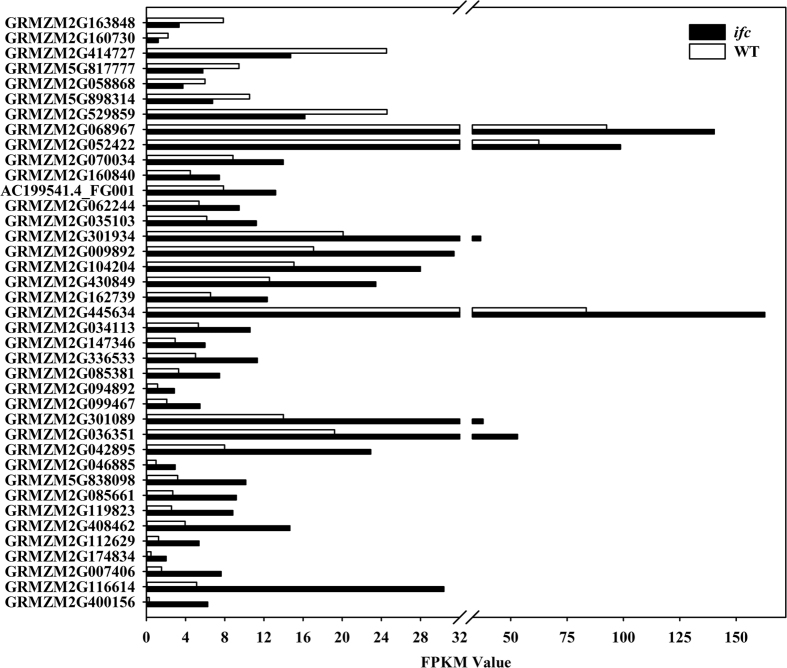
FPKM values for all 39 validated genes. “//” represents 32–33. WT, wild-type; *ifc*, *incompletely fused carpels*.

To confirm the differential expression identified by RNA-seq analysis, we used qRT-PCR to validate the expression levels of 39 transcripts and of which 37 (94.9%) showed expression level differences consistent with the transcriptomic data (Figs 4 and 5). Additionally, 24 genes with differential expression at a fold change of 2 or bigger than 1.5 were also confirmed by qRT-PCR among all the 39 selected genes (Fig. 5). The results showed that 22 genes had a significant difference based on one-tailed *t*-test analysis, which indicated that the differences in gene expression were well confirmed by qRT-PCR, as shown in Figs 4 and 5.

### Functions of Differentially Expressed Genes Between Wild-type and Carpel Fusion Deficient Ovaries

**Figure 6:**
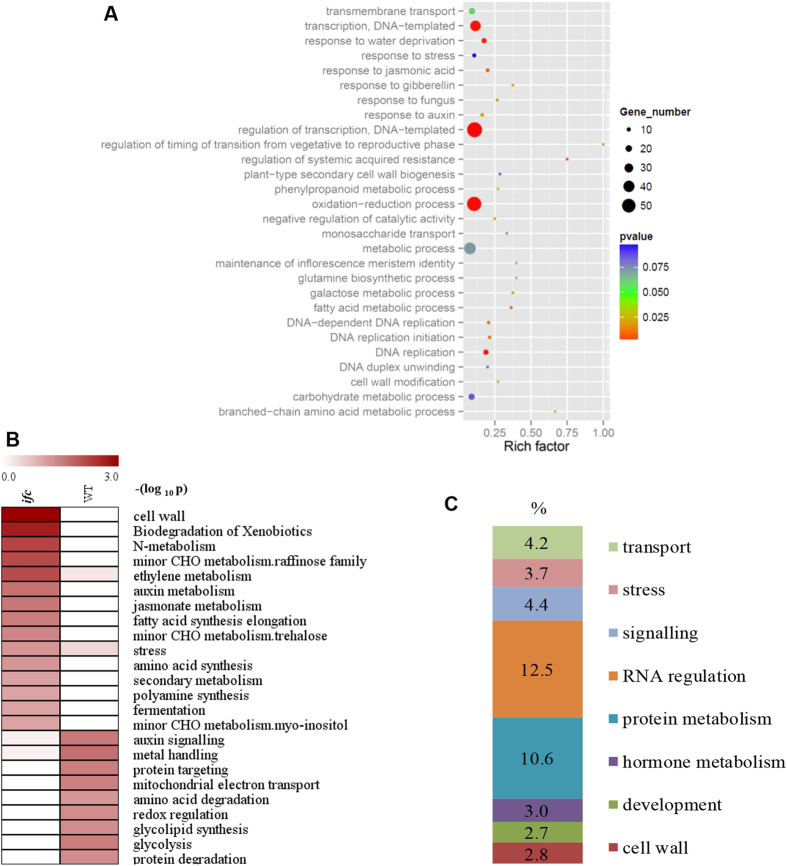
Functional category enrichment analysis of the differentially expressed genes between WT and *ifc* ovaries. (**A**) The enriched GO “biological processes” among the up-regulated genes in *ifc* ovaries. Rich factor represents the ratio of the number of differentially expressed genes over the total number of genes in the GO item. For the details of the GO enrichment analysis, see Table S9. (**B**) MapMan enrichment analysis of the up-regulated genes in *ifc* and WT ovaries. (**C**) Summary of the nine primary functional categories obtained after MapMan enrichment of all differentially expressed genes identified between WT and *ifc* ovaries. The percentage represents the ratio of the number of differentially expressed genes in each category over the total number of all enriched differentially expressed genes in the MapMan analysis. For complete information about the MapMan enrichment analysis, see Table S10. WT, wild-type; *ifc*, *incompletely fused carpels;* CHO, Carbohydrate.

A Gene Ontology (GO) term enrichment analysis of the differentially expressed genes between WT and *ifc* mutant ovaries was performed. The represented biological processes corresponded to the regulation of transcription, transport, and metabolic processes (Fig. 6A; Figure S4; Table S9). Among the genes that were up-regulated in the *ifc* mutant ovary, the categories of DNA replication, nucleus, cell wall, and DNA binding were overrepresented, suggesting that cell division, elongation, and expansion were active in *ifc* ovaries (Fig. 6A; Figures S5 and 6; Table S9). Genes associated with the stress response and water deprivation were also up-regulated in *ifc* mutant ovaries.

Genes that were down-regulated in *ifc* mutant ovaries showed enrichment in the functions of DNA biological processes that are primarily involved in DNA binding, regulation of transcription, and metal ion transport (Figure S4; Table S9). One down-regulated gene, GRMZM2G160730, which encodes a protein related to the specification of organ identity, was also included. The protein has two predicted AP2 domains, and it has been reported that genes within this character in AP2 family are involved in gynoecium development^26^,^34^.

We used MapMan^35^ to explore the differential biological pathways between the WT and *ifc* ovaries. Twenty-four biological pathways were partitioned between WT and *ifc* mutant ovaries (Fig. 6B). Interestingly, genes involved in the hormone metabolism of ethylene, auxin, and jasmonate were overrepresented in *ifc* ovaries, whereas genes related to auxin signaling were overrepresented in WT ovaries (Fig. 6B,C; Table S10), which indicates that the complex hormone regulation network is altered between WT and *ifc* ovaries. Together, these data demonstrate that the major differences in the *ifc* mutant ovary are reflected in part by transcription regulation and hormone signaling networks.

### Differential Expression of TFs between the WT and *ifc* Ovary

**Figure 7:**
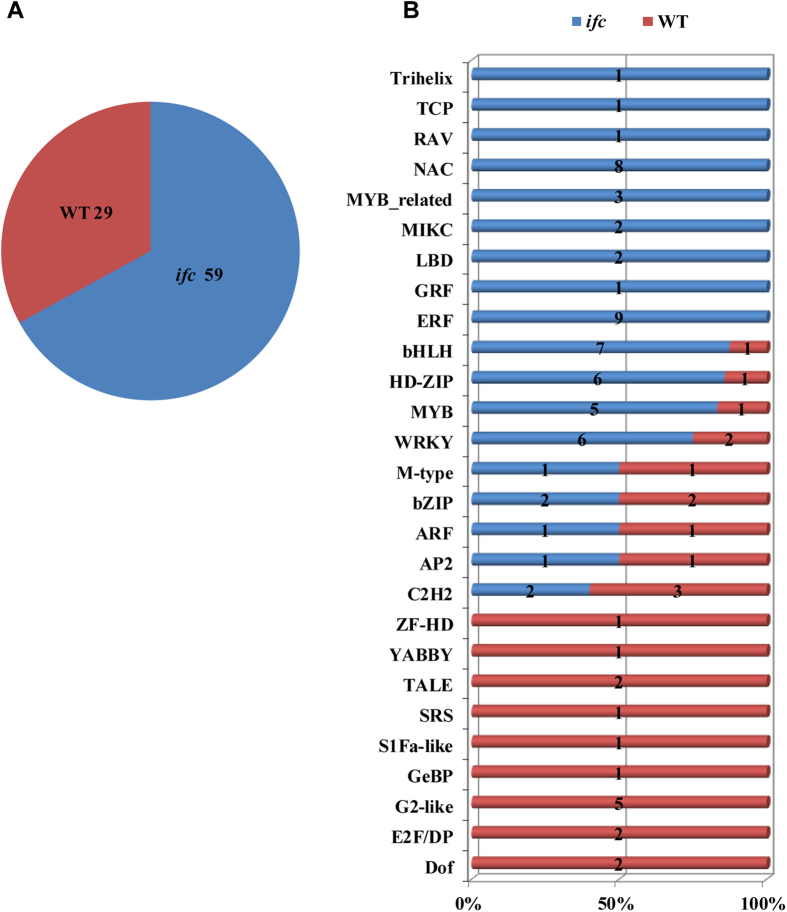
Distribution of differentially expressed TF families between WT and *ifc* ovaries. (**A**) Up-regulated TF genes in WT (red) and *ifc* (blue) ovaries. (**B**) The exact number of up-regulated genes in each family identified in WT (red) and *ifc* (blue) ovaries is shown. For detailed TF-related gene information, see Table S11. WT, wild-type; *ifc*, *incompletely fused carpels.*

To investigate potential regulators involved in ovary wall formation, we focused our analysis on TF genes exhibiting differential expression between WT and *ifc* ovaries. Collectively, among the 1,341 expressed TFs detected in association with the two phenotypes, 88 were differentially expressed (with FPKM > 2 in at least one of the samples), which were classified into 27 TF families (Fig. 7A,B; Table S11) according to the Plant Transcription Factor database v3.0^36^. Overall, two-thirds of the TFs were up-regulated in the *ifc* mutant ovary (Fig. 7A; Table S11). Statistical analysis according to the hypergeometric distribution showed that 10 TF families were significantly differentially expressed, among which the WRKY, HD-ZIP, bHLH, ERF, RAV, and NAC families were significantly up-regulated in the *ifc* ovary, while the G2-like family was significantly down-regulated (*p *< 0.05, hypergeometric test). While the C2H2, AP2, and MYB TF families were also significantly differentially expressed (*p *< 0.05, hypergeometric test) (Table S12). Two members of the differentially expressed NAC family, GRMZM5G898290 and GRMZM2G009892, shared 81.75% and 80.16% identity, respectively, with the AtCUC2 protein of *Arabidopsis* in the conserved NAM domain at the amino acid sequence level. These results suggest putative functions of the two NAC genes in carpel development given that overexpression of *AtCUC2* prevents congenital carpel fusion in *Arabidopsis*^15^.

Basic helix-loop-helix (bHLH) TFs have been associated with the regulation of diverse processes, including organ morphogenesis, anthocyanin production, trichome development, and light signaling through phytochromes^10^. In this study, eight members of the bHLH family of TFs were found to present differential expression, and seven members were up regulated in the *ifc* mutant ovary. One of these TFs, GRMZM2G094892, showed a predicted amino acid sequence similarity of 78.12% in the HLH superfamily DNA binding domain with the HECATE1 protein, a known regulator of carpel development in *Arabidopsis*^37^. The up-regulation of bHLH TFs in the *ifc* mutant ovary is consistent with their reported role in initiating gynoecium development in *Arabidopsis*^37^.

The TFs C2H2 and MYB were also differentially expressed between *ifc* mutant ovaries and WT ovaries, most of which are associated with floral organ-specific transcriptional regulation^38^,^39^. In this study, we identified GRMZM2G160840 as showing 77.66% amino acid sequence identity to the AtMYB21 protein in the conserved DNA binding domain, whose ectopic expression causes malformation of various flower tissues, including hooked and twisted carpels^38^. One C2H2 family member, GRMZM2G058868, a homolog of *Arabidopsis* ZFP10, showed 88% similarity to the SUPERMAN protein in the single zinc finger domain.

These results suggest the transcriptional regulation of gene expression underlying the carpel wall fusion event. However, we note that these predicted TFs require further experimental validation, such as genetic regulation network analysis and more precise mapping and functional studies.

### Differentially Expressed Genes Participate in Hormone Responses and Biosynthesis

**Figure 8:**
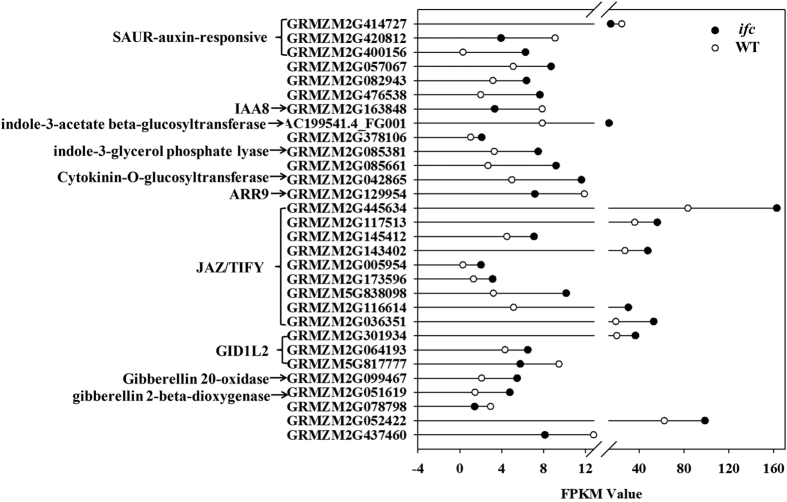
Differentially expressed genes related to hormone signaling and metabolism, with solid and hollow circles indicating gene FPKM values in *ifc* and WT ovaries, respectively. “//” indicates FPKM values from 12.8 to 13. WT, wild-type; *ifc*, *incompletely fused carpels.*

Our transcriptome analysis revealed several genes that participate in biosynthesis, signaling, and the response components of hormonal pathways. Auxin is a major controlling signal that synchronizes flower development and controls carpel marginal tissue formation during gynoecium morphogenesis^20^ in *Arabidopsis*. Genes involved in auxin biosynthesis and responses were represented in the list of differentially expressed genes. Two members of the SAUR-auxin-responsive (SAUR) families were down-regulated, and one was up-regulated in the *ifc* mutant ovary (Fig. 8). One Auxin (Aux)/Indole-3-Acetic Acid (IAA) family member, IAA8, whose homolog is involved in hormonal signaling pathways in *Arabidopsis*^21^, was also down-regulated. In contrast, four key genes involved in auxin biosynthesis were up-regulated. The expression levels of indole-3-glycerol phosphate lyase and indole-3-acetate beta-glucosyl transferase, which have been described in auxin conjugation^40^, were up-regulated (Fig. 8).

Additionally, Cytokinin-O-glucosyltransferase, an enzyme that catalyzes the formation of glucosides from CK, was down-regulated in the *ifc* mutant ovary. Another differentially expressed gene homolog to *Arabidopsis* response regulator 9 (ARR9), a member of the type-A ARR proteins, was down-regulated in the *ifc* mutant ovary (Fig. 8). Type-A ARRs exhibit short C-terminal domains and are rapidly transcriptionally up-regulated by CK treatment^41^.

Genes involved in gibberellin biosynthesis and responses and genes encoding JASMONATE Zim-domain (JAZ)/TIFY proteins were also present in the list of differentially expressed genes. JAZ proteins belong to the larger family of TIFY proteins^42^. Recently, JASMONATE ZIM-domain (JAZ) proteins have been identified as repressors of Jasmonic Acid (JA) signaling^43^. Expression of JAZ1/TIFY10A can be induced by JA, which is also an early auxin-responsive gene^44^. In this study, nine differentially expressed homologs of *Arabidopsis* JAZ/TIFY were found to be up-regulated. Additionally, two GID1L2 gibberellin receptor genes were up-regulated in the *ifc* mutant ovary, while one was down-regulated. Gibberellin 20-oxidase and gibberellin 2-beta-dioxygenase, which are enzymes involved in the biosynthetic pathway for gibberellin, were also up-regulated (Fig. 8). Together, these results indicated the complicated roles played by hormones in the *ifc* mutant ovary, especially in the case of auxin and CK hormones, which may play an important role in aberrant carpel fusion in *ifc* ovaries.

## Discussion

### Incompletely Fused Carpels Affect the Integrity of the Maize Kernel Pericarp, Resulting in a Gap on the Top

According to botanical definition, the maize kernel is classified as a caryopsis, a complex tissue in which maternal and filial tissues follow distinct, but coordinated developmental programs. The pericarp consists of the transformed ovary wall covering the seed. Interestingly, the *ifc* mutant exhibited an incomplete kernel surface structure. Initially, we suggested either an incomplete ovary wall or overgrowth of nucellus tissue that outgrows the ovary wall as possible explanations for the *ifc* phenotype. To confirm which of these possible developmental changes gave rise to this mutant and to determine the emergence of the phenotype, we examined the development of the florets, pistils, and kernels through scanning electron microscopy (SEM) (Fig. 2B–K) and based on histology (Fig. 2L–O).

The *ifc* kernel had a gap on the surface with a distinct structure transition. The gap on the top of the mature kernel was located at the same position as the stylar canal, which is close to the silk and back to the embryo (Figs 1G,I and 2A). Through serial observations, we found that the gap on the top of the ovary was first observable before the extruding nucellus (Fig. 1H), and the protruding nucellus tissue in the *ifc* mutant ovaries may therefore not be due to overgrowth of the tissue, but rather, to being free from carpel wall constraints. These findings were consistent with the hypothesis that the formation of the gap in the *ifc* ovary wall is derived from the open stylar canal due to its incompletely fused carpels.

Histological examination (Fig. 2L–O) via vertical sectioning of the ovaries and kernels revealed that the exposed seed coat (Fig. 2M–O) in the *ifc* ovaries lacked pericarp wrapping. However, the structure of aleurone layer, a part of the endosperm, was not affected, and this one-celled layer could be clearly observed under the thin, suberized seed coat (Fig. 2O). The pericarp is derived from the carpel wall/ovary wall, and along with defects in growth and development, the *ifc* kernels lacked pericarp wrapping on the gap surface. Additionally, the position of the defect fit well within the stylar canal (Fig. 1A,D,F; Fig. S1). Therefore, these results were consistent with the observations from SEM analysis, indicating that the gap on the top of the *ifc* kernel may be an open stylar canal resulting from incompletely fused carpels. The stylar canal is an opening through the ovary wall (pericarp) and has been reported as location of infections because it allows fungal pathogens to enter unwounded kernels, with the severity of infection depending on the degree of opening of the canal^5^. In the *ifc* ovary, the length of the silk derived from the two abaxial carpels (Table S1) displayed no difference from the length of silk in the wild-type ovary. Both the length of the silk in the *ifc* ovary and the ability to undergo fertilization (assessed based on the development of *ifc* kernels on different days after pollination) were normal (Fig. S1). Nevertheless, the stylar canal was open in the *ifc* ovary, as shown in Fig. 2C, which showed the *ifc* ovary and its wild type on the same position in the same ear when carpel fusion defects could be morphologically distinguished. Compared with the normal ovary, the *ifc* ovary exhibited normal length of the silk derived from the fusion of abaxial carpels but presented an open stylar canal resulting from the fusion of the third carpel with the abaxial carpels. In contrast to the closed stylar canal of the wild-type ovary, it is clear that the open stylar canal of the *ifc* ovary is due to insufficient growth of the third carpel. This conclusion is consistent with previous findings that the aberrant development of the third carpel has no effect on the other two carpels^3^. For example, the third carpel gives rise to two ovules, but the other two carpels can normally develop into silk, which grows indeterminately to form the longest stigma of any species^3^.

Maize WT and *ifc* ovaries can be distinguished by their capacity to produce completely fused carpels^4^. Interestingly, kernels on the same ear displayed different pericarp integrities. In the WT kernel, the pericarp was intact with a closed stylar canal, while in the *ifc* ovary, there was a gap at the top of the kernel, resulting from incompletely fused carpels. One explanation for the difference between the WT and *ifc* mutant ovaries is differential gene expression. To better understand the molecular basis of this variation, we performed transcriptome analysis at a stage when the incompletely fused carpels were just beginning to be distinguishable.

### Analysis of Transcriptional Changes Outlining the Main Molecular Processes in the *ifc* Ovary

We performed deep transcriptomic surveys of the WT and *ifc* ovaries in maize using RNA-seq technology. The total number of expressed mRNAs was approximately 35,000, which is equivalent to the number of identified in a recent profiling analysis of maize kernel samples from 8–12 DAP^45^. These similar numbers suggest that our sequencing procedure was sufficient to thoroughly capture nearly all genes expressed in maize kernels.

A total of 877 differentially expressed genes were detected in the present study (Fig. 3C; Table S7), some of which might form a complex transcriptional regulatory network that plays an important part in the development of carpels, as suggested previously for *Arabidopsis*^15^,^46^. In the *ifc* mutant ovary, the exposed nucellus produced from the ovule primordium without constraint by the carpels has the potential to become a large mass of tissue and be extruded from the surrounding carpel wall (Fig. 1H,J). In the *ifc* ovary, a number of differentially expressed genes associated with cell wall biosynthesis were overrepresented. Cell divisions leading to nucellus meristems might provide a large number of new meristematic cells, which is a process that requires enhanced cell wall biosynthesis. Stress-related genes were also overrepresented among the differentially expressed genes. The major function of the endocarp, the innermost layer of the pericarp, is water transport^47^. The incompletely fused carpelsincompletely fused carpels created a defective ovary wall, suggesting that the normal circulation and supply of water via the ovary wall might be insufficient. This could lead to induction of stress-related genes in the *ifc* mutant ovary. Moreover, the functional category of transport was overrepresented among the differentially expressed genes. It is not surprising given that the pericarp of the *ifc* ovary is incomplete, and there is a gap at the top of the kernel. One of the functions of the pericarp is transportation of mineral nutrients and small molecules^47^, and an incomplete ovary wall might therefore affect the normal transport of mineral nutrients and small molecules in the *ifc* ovary. This suggests that the differentially expressed genes in this category are a result of changes in upstream gene expression, giving rise to the observed phenotypic variation.

### Transcription Factor Expression Analysis Indicates Potential Gene Regulation in the Carpel Fusion-Deficient Ovary

In *Arabidopsis*, at least 86 genes are involved in the processes of carpel margin meristem and carpel marginal tissue development, and at least 57 (65%) of these genes with corresponding mutant phenotypes in the gynoecium are TFs^1^. In maize, however, the functions of TF genes in controlling carpel marginal tissue development and carpel fusion remain largely elusive. Hence, our RNA-seq dataset might serve as a resource for developing and testing hypotheses regarding the regulatory networks that govern carpel fusion, for example, through the analysis of TFs with differential expression levels. The differential expression of TF genes suggests a role of some of these genes in specifying carpel margin fusion development. For instance, five members of the NAC, bHLH, C2H2, and MYB TF families were found to accumulate preferentially in the *ifc* ovary. Mutation of these TFs homologs in *Arabidopsis* resulted in carpel fusion defects^15^,^37^,^38^,^39^, implying that these five genes show a high probability of regulation associated with carpel fusion in maize. The genes encoding these TFs are excellent candidates for future functional genomics studies to dissect carpel fusion development in maize.

It is noteworthy that a maize homolog of AP2 was found to be down-regulated in the *ifc* ovary. *AP2*, a class A gene, plays a central role in establishing the floral meristem, specifying floral organ identity, and regulating floral homeotic gene expression in *Arabidopsis*^26^. In addition, in strong *ap2* mutants, carpel fusion is often defective^48^. In *Arabidopsis*, the specification of carpel identity is achieved through at least two separate pathways. Both pathways are negatively regulated by class A genes^49^. The *SPT* gene, a component of the AG-independent pathway^49^, has been shown to interact with three HECATE1 proteins in yeast two-hybrid experiments^37^. The transcription factor GRMZM2G094892, which is 78.12% identical with HECATE1 in the amino acid sequence of conserved domains, was up-regulated in the *ifc* ovary. Therefore, we propose that GRMZM2G094892 is one component of the AG-independent pathway and is negatively regulated by *AP2* in maize.

### Expression of Hormone-Related Genes in the Carpel Fusion-Deficient Ovary

Analysis of the expression of genes involved in the biosynthesis and signaling of different plant hormones provided evidence that these genes also play an important role in normal carpel marginal tissue development. Two auxin-responsive genes were differentially expressed (Fig. 7B). Mutations in genes involved in ARF TFs have been shown to lead to alterations in gynoecium development and patterning, including clear defects in marginal tissue^20^,^50^. All *JAZ/TIFY* genes, including six *JAZ1/TIFY10A* genes, were preferentially enriched in the *ifc* mutant ovary. The expression of *JAZ1/TIFY10A* is auxin-inducible and independent of the JA signaling pathway but is controlled by the ARF signaling pathway^44^. However, genes related to auxin biosynthesis were also up-regulated in the *ifc* mutant ovary, implying complex interactions between these genes during carpel fusion-deficient ovary development.

We also found evidence of the involvement of a different gene family associated with GA biosynthesis and signaling. Transcriptional repressors of GA signaling result in a moderate increase in style length and stigma width, which is related to cell elongation, but not to carpel marginal tissue development^23^. Our data regarding GA metabolism are consistent with previous reports showing induction of GA biosynthesis through increasing *GA20ox* transcription^51^. GA and auxin signaling are known to interact during fruit set and development, as observed in this study from the up-regulation of GA biosynthesis corresponding to auxin, and it is thought that auxin activity induces GA biosynthesis by increasing *GA20ox* transcription^51^. We suggest high possibility of relevance between up-regulation of *GA20ox* transcription and gene encoding auxin biosynthesis in the *ifc* ovary.

Finally, the gene encoding CK O-glucosyl transferase (the enzyme that inactivates CK) was up-regulated in the *ifc* mutant ovary. Experimental evidence indicates that the CK pathway is regulated by the TF SHOOT MERISTEMLESS^52^, and mutations of this TF lead to conspicuous defects in gynoecium morphology in *Arabidopsis*^53^. It has been demonstrated that a differentiated state requires high levels of GA and low levels of CK, and the hormonal balance appears to be controlled by the KNOX proteins^52^. The diverse expression profiles of genes related to the hormone response and biosynthesis identified in this study might constitute the basis for the diversification of hormone signaling pathways in carpel fusion development.

In summary, our analysis of RNA-seq data and morphological, and histological analyses offer a valuable resource for identifying candidate genes involved in carpel fusion. We found that the deficiency on the top of the kernel is caused by incomplete fusion of the carpels. We illustrated the key transcription regulators and hormonal interactions underpinning this development, adding insights into the biological and cellular processes. However, the nature of the key regulatory factors remains to be explored. In particular, future studies should aim to characterize TFs and hormonal regulation functionally for a deeper understanding of carpel fusion in maize by integrating genetic, biochemical, and cytological approaches.

## Materials and Methods

### Plant Materials and Growth

*Zea mays* L. (maize) inbred Yu-A474 plants were grown at a research farm at Henan Agricultural University, Henan Province, China (34°53′N, 113°35′E, 94 m altitude) under non-stressed conditions between June and October 2014. Yu-A474, an inbred line belonging to the Reid germplasm, is the female parent of the commercial hybrid YD603. It shares close relatives with zheng58, the female parent of another hybrid (ZD958) that is widely grown in China. Yu-A474 exhibits strong heterosis with Yu-B469, which is a descendants of Huangzaosi and has a normal kernel pericarp. The meteorological conditions in Henan province, including the average temperature and length of the light, are shown in Table S13. Before pollination, the ear shoots of Yu-A474 were covered before silking to prevent fertilization. The ovaries were collected when the carpel wall was first observed to deviate from normal development. Biological replicates were obtained from three different ear rows, and each set of ovaries was generated on the same ear and during the same time of the day. In all cases, the ovaries were selected on the middle of the ear where a normal ovary was adjacent to a captured *ifc* mutant ovary. The ovaries were isolated by removing the glumes, lemma, and palea and then pinching off the ovary at the base using a pair of forceps. The ovaries were then immediately frozen in liquid nitrogen and stored at −80 °C until RNA extraction. Three biological replicates of both phenotypes were used for RNA-seq. The triplicate samples were subsequently used for quantitative RT-PCR (qRT-PCR) analyses, with each assay containing three technical replicates.

### Measurement of Silk Length

A total of 30 selected ears, whose ear shoots were covered before silking to prevent fertilization, were harvested at silking from plants showing consistent growth. Each ear was divided into 3 parts: basal (the bottommost 3–10 circles of ovaries), middle (between the basal and apical parts), and apical (the topmost 3–10 circles of ovaries). Altogether, 10–15 silks from four sides in each part of the ear were measured. The one-tailed *t*-test was used to detect significant differences.

### Microscope Sections

For histological analysis, maize ovaries were collected at silking, when it was possible to observe the carpel wall deviating from normal development, after repeated observation and discrimination. Maize kernels were harvested after 2, 3, 4, 6, 8, 10, 12, 15, 18, 21, 24, 27, 30, and 35 days of pollination, representing a wide range of pericarp changes noted during visual observations. Briefly, after fixation in 5 mL of formaldehyde, 5 mL of acetic acid, and 90 mL of 50% ethanol (FAA) at 4 °C for 24 h, the samples were dehydrated through an increasing ethanol series (10–100%), infiltrated and embedded in paraffin (Sigma), then sliced into 8-μm-thick sections using a rotary microtome (Leica). The sections were subsequently stained with Safranin-Fast Green (Safranin O, Sigma). After staining, the sections were dehydrated through a reverse water/ethanol/xylene series. Permanent slides were mounted with synthetic resin (Entellan). Observations were carried out with a Nikon 80I optical microscope (Tokyo, Japan), and images of the maize ovaries and kernels were collected using a Nikon SSZ1500 dissecting microscope (Tokyo, Japan).

### Scanning Electron Microscopy

Young, primary ears and florets were dissected under a stereomicroscope. The organs in the outer whorls, including the lemma, palea, and lodicule, were removed by “cutting” them with forceps to view the innermost pistil. For observation of the aleurone layer in WT ovaries, the pericarp tissues were micro-dissected using a sharp blade and anatomical tweezers. The samples were fixed in 2% glutaraldehyde in 0.2 M sodium phosphate buffer (pH 7.2) at 4 °C for at least 24 h. The slides were then rinsed in 0.2 M sodium phosphate (pH 7.2) and dehydrated in a graded ethanol series at 4 °C. Finally, the slides were immersed in tertiary butyl alcohol and stored in a refrigerator at 4 °C. Thereafter, the samples were placed in the bell jar of a vacuum evaporator overnight and evacuated with a rotary pump at 4 °C. After sublimation of the frozen tertiary butyl alcohol in the vacuum, the samples were completely dried. Finally, the samples were mounted on SEM stubs with double-sided tape, sputter-coated with gold, and viewed in a Hitachi S-3400NIISEM using accelerating voltages of 5–10 kV.

### RNA extraction, cDNA Synthesis, and Amplification

Total RNA for RNA-seq and qRT-PCR analyses was extracted from maize ovaries using TRIzol reagent (Invitrogen), following the manufacturer’s instructions. Residual genomic DNA was removed by on-column DNase I treatment (Qiagen), and RNA was further purified by passaging through RNAeasy columns (Qiagen). For each of the six samples, ribosomal RNA (rRNA) was removed from the total RNA samples using Ribo-Zero™ Magnetic (Epicentre). cDNA synthesis and amplification of each replicate were performed according the manufacturer’s protocols (Illumina) with minor modifications. The integrity and quality of the RNA and amplified cDNA were checked using a Bioanalyzer 2100 (Agilent).

### Library Construction and Sequencing

For the construction and sequencing of RNA-seq libraries, approximately 10 μg of rRNA-depleted RNA was used to construct strand-specific cDNA libraries with a mean size 400 ± 50 bp, following a previously described procedure^54^ for deep sequencing according to the standard Illumina protocol. Paired-end sequencing (2 × 125 bp) was performed on the Illumina HiSeq-2500 platform. After the raw reads were generated, low-quality reads (reads containing sequencing adaptors; reads containing sequencing primers; nucleotides with a Q quality score lower than 20) were trimmed using the Trimmomatic program^55^. The quality of the raw reads was checked using the FastQC (http://www.bioinformatics.babraham.ac.uk/projects/fastqc/).

### Data Mapping and Analysis

RNA-seq reads were mapped against the maize reference genome version 3 (B73 RefGen_v3)^29^ using TopHat2^56^. Intron length was set to 45–40,000 nucleotides, while the maximum number of mismatches per read was set to 3. To eliminate the effect of read mapping in intergenic and/or repeated genomic regions, the reads that were mapped to exonic regions were extracted using the intersect function of BEDTools v2.17.0^57^ and were provided as input to Cufflinks v2.1.1^31^ for normalization and estimation of gene expression levels.

Transcript assembly was performed using Cufflinks, and differentially expressed genes were identified with Cuffdiff using the multi-mapped read correction, fragment bias correction, and quartile normalization options^30^,^31^. To quantify the expression of genes and transcripts, gene expression levels were reported as FPKM values. The upper and lower boundary FPKM values (FPKM_conf_hi and FPKM_conf_lo, respectively) from Cufflinks for the 95% confidence interval of each gene were used. When the FPKM_conf_lo was greater than zero, a gene was defined as being expressed in a sample. The information related to maize genome annotation used in this study was obtained from Ensembl Plants (plants.ensembl.org, release 28).

The reproducibility of the data between the triplicate samples from WT and *ifc* ovaries was quantified through SCC analysis^32^. Log2-transformed FPKM values [log2 (FPKM + 1)] of the expressed genes were used as the input for the SCC analysis. According to the high correlation of gene expression profiles among the biological replicates, exonic reads were pooled to create a union for both sets of triplicates, and FPKMs were recalculated for the two pooled samples. The cutoff for differential gene expression was set at a fold change greater than 1.5 and a *P*-value < 0.05.

### Gene Annotation and Functional Enrichment Analysis

The locus information and functional annotations for maize genes were based on information from Ensemble Plants. TF family members were classified according to the Plant Transcription Factor Database v3.0^36^. The putative functions of the genes of interest were redefined and inspected manually using BLAST searches and database annotations. Gene Ontology (GO) annotations and enrichment analyses of the genes were performed using Blast2GO (Fisher’s exact test, *P *< 0.05) software (http://www.blast2go.com/). GO annotations for maize genes were obtained from Gramene (gramene.org, release 40). MapMan annotation was performed according to Thimm^35^. For each functional enrichment, only the protein-coding genes (i.e., transposable elements, microRNA genes, and pseudogenes were excluded) were subject to analysis, with modified MapMan bins as the functional categories. Fisher’s exact test (*P *< 0.05) was used to investigate the significance level of each category.

### Real-Time RT-PCR Analysis

To validate the RNA-seq results, real-time PCR was performed using SYBR Green (Applied Biosystems) and the 7900 HT Sequence Detection System (Applied Biosystems), followed by real-time PCR analysis. The absence of contaminant genomic DNA was confirmed in the reactions using RNase-free, DNase-treated RNA samples as a template. Reverse transcription and qRT-PCR were performed as previously described^58^. *EF1a* (gene ID: GRMZM2G153541) was found to show the lowest level of variation and was therefore used as an internal reference gene to normalize the amount of template cDNA. These values were then compared with FPKM estimates to determine whether our original samples for RNA-seq were reliable. Three biological replicates were conducted via quantitative RT-PCR. The primers used in these experiments are listed in Table S14. The one-tailed *t*-test was employed to compare the significance of the differences in the gene expression data obtained through RT-PCR.

## Additional Information

**Accession codes:** The raw data have been deposited at the National Center for Biotechnology Information Gene Expression Omnibus. The following link has been created to allow the review of record GSE76800 while its status is private: (http://www.ncbi.nlm.nih.gov/geo/query/acc.cgi?token=gbufooyabjgbxex&acc=GSE76800).

**How to cite this article**: Li, H. *et al*. Differential morphology and transcriptome profile between the *incompletely fused carpels* ovary and its wild-type in maize. *Sci. Rep.* **6**, 32652; doi: 10.1038/srep32652 (2016).

## Supplementary Material

Supplementary Information

Supplementary Table S4

Supplementary Table S6

Supplementary Table S7

Supplementary Table S9

Supplementary Table S10

Supplementary Table S11

Supplementary Table S14
