# Supplementary material for: Differential morphology and transcriptome profile between the incompletely fused carpels ovary and its wild-type in maize
Source: Sci Rep. 2016 Sep 2;6:32652. doi: 10.1038/srep32652 (PMC5009309; doi:10.1038/srep32652)
Supplement: Supplementary Information [file srep32652-s1.pdf]

Supplementary information

Differential morphology and transcriptome profile between *incompletely fused carpels* ovary and its wild-type in maize

Hongping Li[1,+], Yufeng Wu[2,+], Yali Zhao[1], Xiuli Hu[1], Jianfeng Chang[1], Qun Wang[1], Pengfei Dong[1], Moubiao Zhang[1], Chaohai Li[1,*]

[1]Agronomy College, Collaborative Innovation Center of Henan Grain Crops, Henan Agricultural University, Zhengzhou 450002, P. R. China

[2]Bioinformatics Center, National Key Laboratory of Crop Genetics and Germplasm Enhancement, Nanjing Agricultural University, Nanjing 210095, P. R. China

*Corresponding author. Tel.:+86 371 63555629; E-mail address: lichaohai2016@sina.com

[+] These authors contributed equally to this work.

Supplemental Figure Legends

Figure S1. Overview of the developmental changes in WT and *ifc* kernels. Bars =1 mm. Numbers represent days after pollination. The WT and *ifc* kernels are from the same ear at same time. Abbreviations: WT, wild-type; *ifc*, *incompletely fused carpels.*

Figure S2. Mapping statistics of the reads aligned to B73 v3 reference genome. A, statistics of valid reads after raw reads filtered. B and C, Statistics of junction annotation in splice events (B) and splice junction (C). WT, wild-type; *ifc*, *incompletely fused carpels*. Biological replicates are indicated as -1, -2 and -3.

Figure S3. Reproducibility of RNA-Seq reads for two triplicates of the wild-type and *ifc* ovaries. Log_2(FPKM+1) of the 35,905 genes expressed in at least one of the 6 sequenced samples are shown as scatter plots and were used as input for the Spearman correlation coefficient (SCC) analysis. The red diagonal line in each scatter plot denotes equal FPKMs between two samples. Abbreviations: WT, wild-type; *ifc, incompletely fused carpel*.

Figure S4. Gene Ontology (GO) enrichment analysis of "biological process" among genes down-regulated in *ifc* ovaries. All the GO terms are significantly enriched. Rich factor, the ratio of significant difference gene number with the total gene number of the GO item. *ifc*: *incompletely fused carpel.*

Figure S5. Gene Ontology (GO) enrichment analysis of "cellular component" among the genes up-regulated in *ifc* ovaries. All the GO terms are significantly enriched. Rich factor, the ratio of significant difference gene number with the total gene number of the GO item. *ifc*: *incompletely fused carpel.*

Figure S6. Gene Ontology (GO) enrichment analysis of "molecular function" among the genes up-regulated in *ifc* ovaries. All the GO terms are significantly enriched. Rich factor, the ratio of significant difference gene number with the total gene number of the GO item. *ifc*: *incompletely fused carpel*.

Figure S1

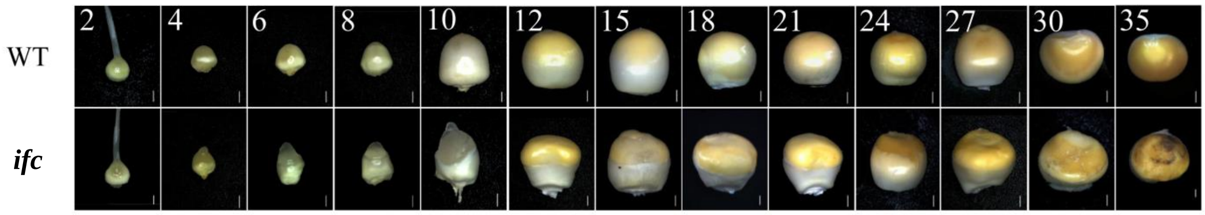

Figure S2

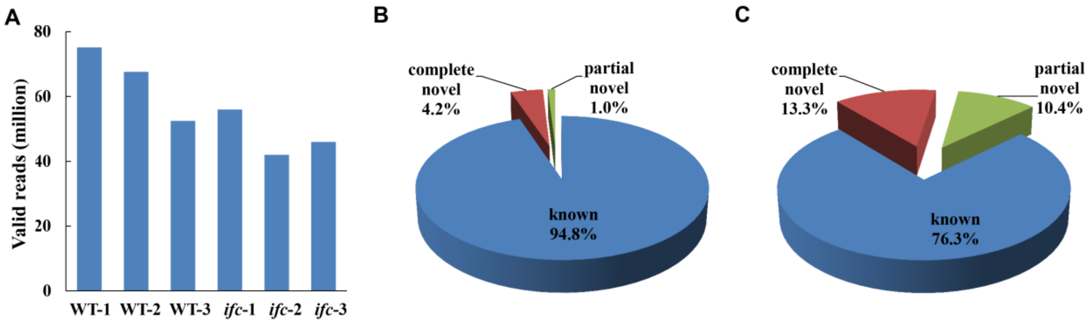
A
Valid reads (million)
0
20
40
60
80
WT-1
WT-2
WT-3
ifc-1
ifc-2
ifc-3
B
complete novel 4.2%
partial novel 1.0%
known 94.8%
C
complete novel 13.3%
partial novel 10.4%
known 76.3%

Figure S3

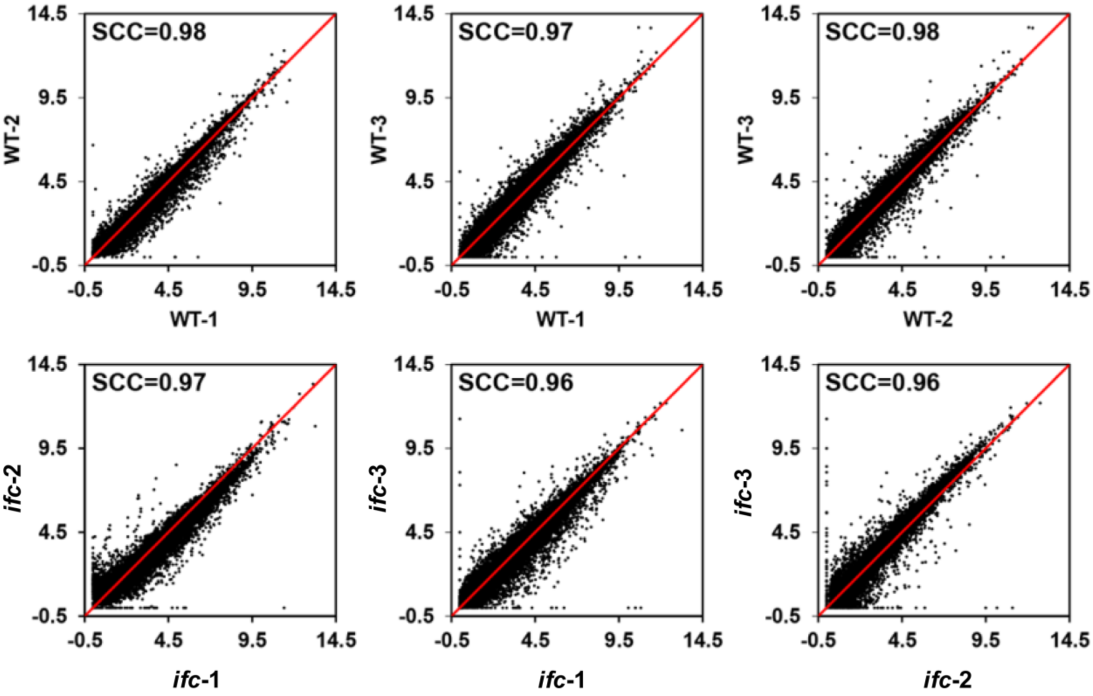
SCC=0.98
WT-2
WT-1
SCC=0.97
WT-3
WT-1
SCC=0.98
WT-3
WT-2
SCC=0.97
ifc-2
ifc-1
SCC=0.96
ifc-3
ifc-1
SCC=0.96
ifc-3
ifc-2
14.5
9.5
4.5
-0.5

Figure S4

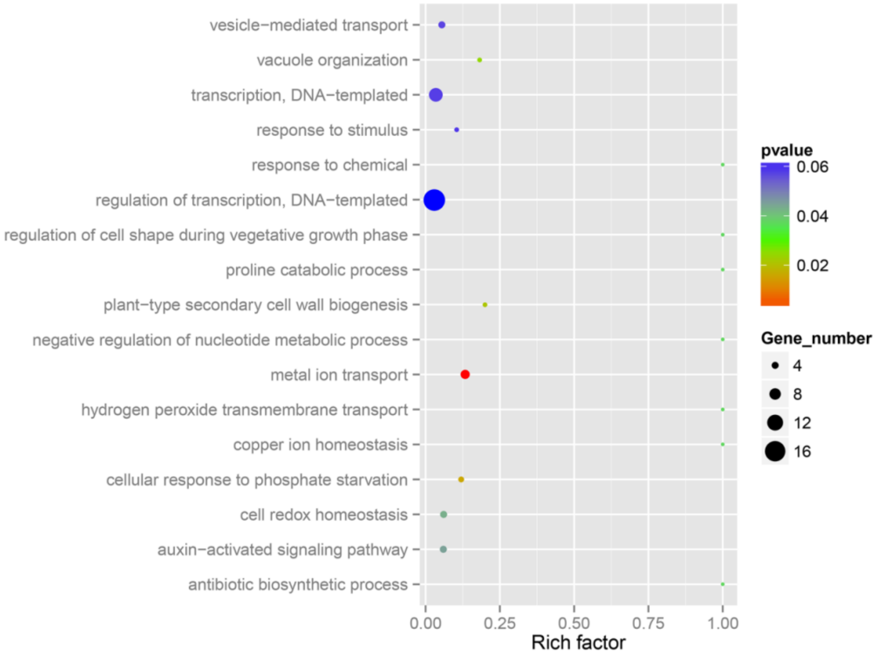
vesicle−mediated transport
vacuole organization
transcription, DNA−templated
response to stimulus
response to chemical
regulation of transcription, DNA−templated
regulation of cell shape during vegetative growth phase
proline catabolic process
plant−type secondary cell wall biogenesis
negative regulation of nucleotide metabolic process
metal ion transport
hydrogen peroxide transmembrane transport
copper ion homeostasis
cellular response to phosphate starvation
cell redox homeostasis
auxin−activated signaling pathway
antibiotic biosynthetic process
0.00
0.25
0.50
0.75
1.00
Rich factor
pvalue
0.06
0.04
0.02
Gene_number
4
8
12
16

Figure S5

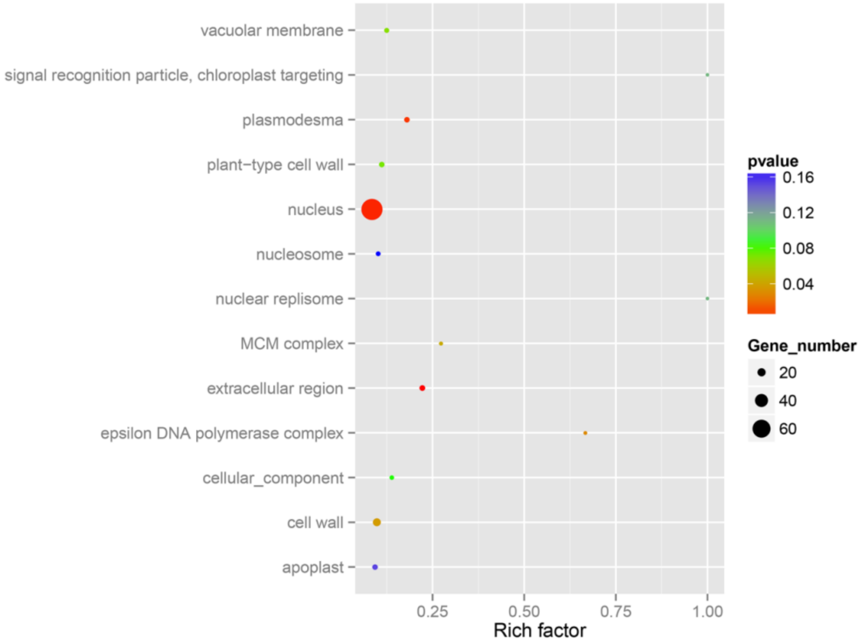

vacuolar membrane
signal recognition particle, chloroplast targeting
plasmodesma
plant−type cell wall
nucleus
nucleosome
nuclear replisome
MCM complex
extracellular region
epsilon DNA polymerase complex
cellular_component
cell wall
apoplast
0.25
0.50
0.75
1.00
Rich factor
pvalue
0.16
0.12
0.08
0.04
Gene_number
20
40
60

Figure S6

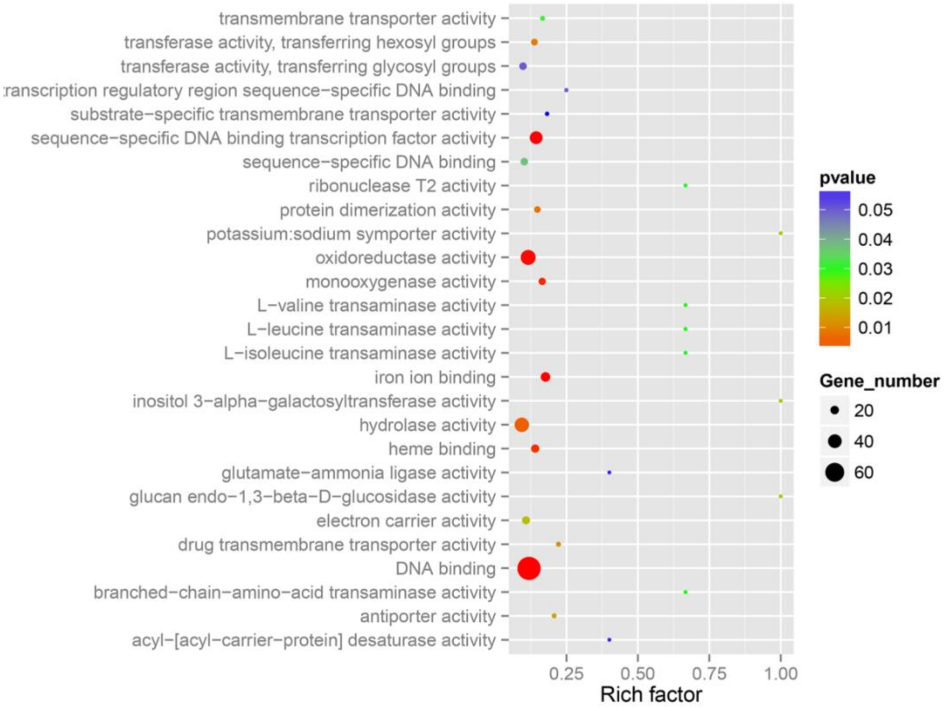

transmembrane transporter activity
transferase activity, transferring hexosyl groups
transferase activity, transferring glycosyl groups
ranscription regulatory region sequence−specific DNA binding
substrate−specific transmembrane transporter activity
sequence−specific DNA binding transcription factor activity
sequence−specific DNA binding
ribonuclease T2 activity
protein dimerization activity
potassium:sodium symporter activity
oxidoreductase activity
monooxygenase activity
L−valine transaminase activity
L−leucine transaminase activity
L−isoleucine transaminase activity
iron ion binding
inositol 3−alpha−galactosyltransferase activity
hydrolase activity
heme binding
glutamate−ammonia ligase activity
glucan endo−1,3−beta−D−glucosidase activity
electron carrier activity
drug transmembrane transporter activity
DNA binding
branched−chain−amino−acid transaminase activity
antiporter activity
acyl−[acyl−carrier−protein] desaturase activity
0.25
0.50
0.75
1.00
Rich factor
pvalue
0.05
0.04
0.03
0.02
0.01
Gene_number
20
40
60

Table S1. Silk length difference of *ifc* and WT ovaries on the same part of the ear

Phenotypes	Basal	Middle	Apical
ifc	21.19±0.78a	15.66±0.38a	11.33±0.43a
WT	20.73±1.14a	16.15±0.71a	12.21±0.24a

WT, wild-type; *ifc*, *incompletely fused carpels*; Values by letter "a" are not significantly different at the 0.05 probability level.

Table S2 Hereditary analysis of pericarp integrity of an ear in different generations in maize

Generation	Total plants/ears	No. of intact pericarp kernel plants/ears		No. of non-intact pericarp kernel plants/ears		Actural ratio	Theoretical ratio	χ^2 value
		Observered	Theoretical	Observered	Theoretical			
P_1 (Yu-A474)	1545	0	0	1545	1545	-	-	-
P_2 (Yu-B469)	1380	1380	1380	0	0	-	-	-
F_0 ($P_1 \times P_2$)	856	0	0	856	856	-	-	-
F_0 ($P_2 \times P_1$)	785	785	785	0	0	-	-	-
F_1 ($P_1 \times P_2$)	2021	2021	2021	0	0	-	-	-
F_1 ($P_2 \times P_1$)	2035	2035	2035	0	0	-	-	-
BC_1 [($P_1 \times P_2$) $\times P_1$]	831	436	415.5	395	415.5	1.10：1	1：1	2.022
F_2 ($F_1 \times F_1$)	790	575	592.5	215	197.5	2.67：1	3：1	2.067

P: parent; F_0: ears of parent plants; F_1: ears of F_1 plants; F_2: ears of F_2 plants; F_2 ($F_1 \times F_1$): [($P_1 \times P_2$) × ($P_1 \times P_2$)]; $\chi^2_{0.05, 1}$=3.84.

Table S3. Summary statistics of RNA-Seq data and mapping.

Samples	Raw Data	Valid reads	Mapped reads	Unique Mapped reads	Multi Mapped reads	PE Mapped reads
WT-1	75,437,372	75,163,058	64,208,485	59,269,143	4,939,342	55,253,912
WT-2	67,864,804	67,618,892	56,445,622	50,521,007	5,924,615	47,272,352
WT-3	52,718,714	52,468,886	43,389,226	40,262,379	3,126,847	36,279,586
ifc-1	56,118,495	55,965,910	46,539,363	43,763,181	2,776,182	39,112,816
ifc-2	42,146,625	41,972,438	33,930,022	30,661,483	3,268,539	27,887,606
ifc-3	46,500,032	45,981,264	33,580,591	31,449,368	2,131,223	23,179,918

WT, wild-type; *ifc, incompletely fused carpels*; Biological replicates are indicated as -1, -2 and -3.

Table S5. Number of genes expressed at different FPKM levels in the six samples.

Samples	FPKM<2		2≤FPKM<10		10≤FPKM<100		FPKM≥100	
	#	%	#	%	#	%	#	%
WT-1	4,818	4.82	8,241	8.24	11,317	11.32	991	1.0
WT-2	5,179	5.18	8,055	8.06	11,099	11.10	1,034	1.0
WT-3	5,119	5.12	7,787	7.79	11,327	11.33	1,134	1.1
ifc-1	4,828	4.83	7,840	7.84	11,434	11.43	1,265	1.3
ifc-2	4,890	4.89	8,929	8.93	10,753	10.75	795	0.8
ifc-3	4,962	4.96	8,001	8.00	11,281	11.28	1,123	1.1

WT, wild-type; *ifc*, *incompletely fused carpels*; Biological replicates are indicated as -1, -2 and -3.

Table S8. Number of genes expressed in each of the samples.

Samples	Protein-coding genes	Transposable elements	Pseudogenes	TFs	miRNAs
WT-1	25,293	2,999	1,573	1,338	17
WT-2	25,331	3,000	1,573	1,339	18
WT-3	25,207	2,990	1,574	1,335	17
ifc-1	25,242	2,992	1,573	1,339	16
ifc-2	25,296	2,997	1,573	1,341	15
ifc-3	25,240	2,994	1,572	1,338	19

WT, wild-type; *ifc*, *incompletely fused carpels*; Biological replicates are indicated as -1, -2 and -3.

Table 12 Hypergeometric distribution analysis of differentially expressed TFs

TF family	Total TFs	No. of differentially expressed TFs	P_d	No. of up regulated TFs	P_u	No. of down regulated TFs	P_d	No. of differentially expressed genes	Total genes
Dof	37	2	0.233	-	-	2	0.233	877	25,369
E2F/DP	12	2	0.055	-	-	2	0.055	877	25,369
G2-like	37	5	0.007	-	-	5	0.007	877	25,369
GeBP	18	1	0.342	-	-	1	0.342	877	25,369
S1Fa-like	2	1	0.067	-	-	1	0.067	877	25,369
SRS	9	1	0.235	-	-	1	0.235	877	25,369
TALE	21	2	0.129	-	-	2	0.129	877	25,369
YABBY	8	1	0.216	-	-	1	0.216	877	25,369
ZF-HD	18	1	0.342	-	-	1	0.342	877	25,369
C2H2	63	5	0.045	2	0.273	3	0.199	877	25,369
AP2	11	2	0.048	1	0.268	1	0.268	877	25,369
ARF	25	2	0.160	1	0.372	1	0.372	877	25,369
bZIP	75	4	0.143	2	0.255	2	0.255	877	25,369
M-type	16	2	0.088	1	0.326	1	0.326	877	25,369
WRKY	58	8	0.001	6	0.011	2	0.276	877	25,369
MYB	82	6	0.041	5	0.090	1	0.164	877	25,369
HD-ZIP	45	7	0.001	6	0.003	1	0.331	877	25,369
bHLH	102	8	0.016	7	0.038	1	0.101	877	25,369
ERF	111	9	0.010	9	0.010	-	-	877	25,369
GRF	9	1	0.235	1	0.235	-	-	877	25,369
LBD	18	2	0.104	2	0.104	-	-	877	25,369
MIKC	25	2	0.160	2	0.160	-	-	877	25,369
MYB_related	63	3	0.199	3	0.199	-	-	877	25,369
NAC	69	8	0.002	8	0.002	-	-	877	25,369
RAV	1	1	0.035	1	0.035	-	-	877	25,369
TCP	27	1	0.374	1	0.374	-	-	877	25,369
Trihelix	40	1	0.351	1	0.351	-	-	877	25,369

P_d: *p* value of differentially expressed TFs; P_u: *p* value of up regulated TFs; P_d: *p* value of down regulated TFs.

Table S13. The meteorological conditions in Henan and Hainan province

Location	Meteorological element	Month					Mean
		6	7	8	9	10	
Henan	Hours of illumination (h)	203.35	171.47	172.85	152.2	162.06	172.39
	Percent of illumination (%)	47.19	38.97	41.65	41.16	46.35	43.06
	Day-night temperature difference (℃)	10.75	8.19	7.87	8.83	10.31	9.19
	Mean temperature (℃)	26.28	27.38	25.95	21.27	15.78	23.33
	Precipitation (mm)	59.59	137.99	126.6	83.56	36.22	88.79
	Mean relative humidity (%)	60.13	75.13	77.42	73.65	65.9	70.45

	Meteorological element	Month					Mean
		10	11	12	1	2	
Hainan	Hours of illumination (h)	195.43	183.92	176.72	180.25	148.84	177.03
	Percent of illumination (%)	53.84	54.55	51.87	52.53	45.9	51.7
	Day-night temperature difference (℃)	5.71	5.8	6.03	5.72	5.41	5.73
	Mean temperature (℃)	26.22	24.36	22.12	21.25	22.25	23.24
	Precipitation (mm)	267.81	68.05	18.54	6.61	14.2	75.04
	Mean relative humidity (%)	77.19	73.23	70.84	74.26	78.16	74.74

These data was downloaded from:

http://www.escience.gov.cn/metdata/page/index.html
